# Evaluation of the Efficacy, Safety, and Adherence to Oral Drug Therapy in Patients with Relapsing–Remitting Multiple Sclerosis

**DOI:** 10.3390/medicina61040762

**Published:** 2025-04-21

**Authors:** Paulius Sėdžius, Dalia Musneckienė

**Affiliations:** Department of Neurology, Lithuanian University of Health Sciences, A. Mickevičiaus Str. 9, LT-44307 Kaunas, Lithuania; dalia.musneckiene@lsmu.lt

**Keywords:** relapsing–remitting multiple sclerosis, teriflunomide, dimethyl fumarate, fingolimod, cladribine, annualized relapse rate, expanded disability status scale, lymphopenia, magnetic resonance imaging

## Abstract

*Background and Objectives:* Selecting appropriate disease-modifying drugs (DMDs) is crucial for optimizing treatment and slowing disease progression in multiple sclerosis (MS). Real-world studies assess drug efficacy and usage in routine clinical practice. Therefore, the goal of this study was to determine the efficacy and safety of oral drug therapy in patients with relapsing–remitting multiple sclerosis and the particularities of adherence to the therapy. *Materials and Methods:* A retrospective and prospective study was conducted at the Neurology Clinic of the Kaunas Clinics of the Lithuanian University of Health Sciences. The medical records of patients with relapsing–remitting multiple sclerosis (RRMS) were reviewed. The retrospective study included 286 patients, and the prospective study included 175 patients. *Results:* The study population included 131 patients on teriflunomide (TFN), 53 on dimethyl fumarate (DMF), 37 on fingolimod (FTY), and 65 on cladribine (CLAD). The overall absolute reduction in the ARR over 4 years of treatment was higher in the second-line (FTY and CLAD) group (−2.00) compared with the first-line (−0.99) group (TFN and DMF). The total EDSS scores of patients who received FTY and CLAD were higher in the second (3.09, *p* = 0.024), third (3.94, *p* = 0.015), and fourth (3.6, *p* = 0.002) years of treatment, compared with the patients of first-line therapy. MRI revealed that the number of contrast-enhancing and new lesions was lower among patients taking second-line drugs in the second year (4.7% and 18.6%, respectively). The worst adherence to the drug therapy due to forgetfulness was observed in the DMF group (30.8%). Lymphopenia was less frequent in the TFN group (93.1%) and more frequent in the FTY group (86.5%) (*p* < 0.001). *Conclusions:* Over four years, second-line patients had greater ARR reduction, fewer MRI lesions, and higher EDSS from year two. DMF showed the lowest adherence, mainly due to patient forgetfulness, while lymphopenia occurred most frequently with FTY.

## 1. Introduction

Multiple sclerosis (MS) is a chronic inflammatory demyelinating degenerative disease of the central nervous system causing physical disability and cognitive impairment [1]. In 2020, the global number of individuals diagnosed with MS reached 2.8 million [2]. Up to 85% of the patients develop the relapsing–remitting (RR) form of the disease, and 54% of them eventually develop secondary progressive disease, which is associated with worsening of the functional status regardless of exacerbations and less effective treatment with DMDs [3,4].

Over the last 20 years, treatment of MS with DMDs has become a standard practice. Since the introduction of beta-interferon, the first injectable MS therapy, numerous new drugs have been developed targeting different pathophysiological pathways of the disease. In 2010, the development of FTY, the first DMDs in a tablet form, offered a more convenient route of administration for some patients. Presently, a number of other oral drugs, including TFN (inhibitor of the mitochondrial enzyme dihydro-orotate dehydrogenase), DMF (causing a shift in the balance of cytokine production via NF-E2-Related Factor 2-Mediated Inhibition), FTY (sphingosine-1-phosphate receptor modulator), CLAD (immune reconstitution therapy), etc., have been developed to modify the course of the disease in RR patients by targeting components of the immune system [5,6]. There are over 15 different approved medications that differ in the route of administration, such as injectable, infusion, or oral forms [7]. The appropriate selection of a disease-modifying drug (DMD) depends on the activity and progression of the disease, as well as on the factors associated with the drug (tolerability, dosage form, efficacy, and cost) [8]. Although currently, there is no therapy to completely stop the disease progression, the prescription of an effective drug might be expected to slow down the progression of RRMS in patients to the secondary progressive stage of the disease. One of the key principles of the therapy is to reduce the frequency of exacerbations and to slow down the development of new, pathological white matter lesions on brain and spinal cord MRI scans. Therefore, the appropriate selection of a first-line (TFN, DMF, etc.) or second-line (FTY or CLAD) DMD of sufficient efficacy, based on the severity of the disease, is essential. High-efficacy disease-modifying therapies are typically given to patients who have failed first-line treatment or patients with high disease activity (based on clinical and imaging findings) [9].

When oral DMDs are prescribed, constant monitoring by means of laboratory tests and imaging examinations is important to avoid side effects. According to studies, FTY patients may experience cardiovascular side effects (atrioventricular block, first-dose bradycardia, and increased arterial blood pressure), increased risk of skin cancer, and progressive multifocal leukoencephalopathy. TFN may cause liver damage, alopecia, and headaches. Administration of DMF may lead to liver enzyme elevations, nausea, diarrhea, and episodes of hot flushes [10]. One of the mechanisms of action of oral drugs is to suppress the immune system by targeting T and B lymphocytes. One of the side effects of these drugs is associated with decreased absolute lymphocyte counts, which is most significant in patients taking CLAD, FTY, and DMF, which not only demonstrates the efficacy of the treatment but also increases the risk of infections [11].

Proper use of the drug is important to achieve the maximum desired effect. Just as is the case for many other therapies used in the treatment of chronic diseases, adherence to the drug therapy regimen is influenced by several factors: frequency of oral DMDs (e.g., administration once or twice a day, a course of treatment), efficacy, tolerability, and side effects [12].

Data registers of MS patients in different countries provide a means of assessing the disease characteristics of MS patients in a particular country, considering the demographic data, the medications prescribed, the frequency of exacerbations, changes in the expanded disability rating scale (EDSS) and magnetic resonance imaging (MRI) scan results, and the results of cerebrospinal fluid and blood tests. The Danish Multiple Sclerosis Registry, established in 1956, is among the oldest databases, with studies covering clinical, social, and financial aspects of healthcare, integrated with data from other Danish registries [13]. Patients admitted to the MS Centre of Kaunas Clinics of the Hospital of the Lithuanian University of Health Sciences undergo a comprehensive examination and are prescribed an appropriate first- or second-line treatment based on the form and severity of the disease and instrumental and laboratory findings. It is therefore important to obtain as many data as possible from the real-life clinical practice to assess the benefits and particularities of the use of drugs in daily practice. Hence, the aim of this study was to determine the efficacy and safety of oral drug therapy in patients with relapsing–remitting multiple sclerosis and the particularities of adherence to the therapy in real-life clinical practice, to build a database and to better understand the efficacy of prescribed medications, particularly when considering drugs with similar efficacy to improve disease outcomes.

## 2. Materials and Methods

### 2.1. Study Design

A retrospective and prospective study was conducted at the Outpatient Unit of the Department of Nervous System Diseases of Kaunas Clinics of the Hospital of the Lithuanian University of Health Sciences. The medical records of patients with RRMS were reviewed. The retrospective study focused on the patients with a confirmed diagnosis of RRMS who received one of the DMDs (TFN, DMF, FTY, or CLAD) as a first-line treatment or as a replacement for another DMD at different time points between 1 January 2016, and 31 May 2024. The prospective study included patients who were prescribed with and continued the use of one of the investigational DMDs before 1 June 2024, and analyzed adherence to the drug therapy by interviewing the patients twice (after 3 and 6 months) by telephone to calculate how many tablet doses (of TFN, DMF, and FTY) were skipped and why and to determine whether the full course of treatment (with CLAD) was completed by 30 November 2024.

Demographic and clinical data were collected during the study. The most recent DMD administered prior to the investigational oral drug was evaluated, with EDSS scores ranging from 0 to 10 changing every 0.5 points in the first year before the DMD and in the 1st, 2nd, 3rd, and 4th year after the administration of the DMD. Exacerbations of the disease (≥24 h of symptomatic exacerbation and occurring within ≥30 days of the last exacerbation) were evaluated by calculating the ARR per patient per year one year before the investigational DMD was prescribed and one, two, three, and four years after the prescription of the DMD. The presence of multiple sclerosis lesions on brain MRI scans in T2-weighted images and contrast enhancement in T1-weighted images were evaluated during the first and second years following the administration of the DMD. The occurrence of lymphopenia (the lower limit of the normal absolute serum lymphocyte count ≥1 ×10^9^/L, as defined by the World Health Organization) in patients treated with the investigational DMD and the side effects caused by the DMD were also analyzed. The study was approved by Kaunas Regional Biomedical Research Ethics Committee (Approval No. 2024-BE10-0004).

### 2.2. Selection of Patients

The study analyzed a population of adult patients (≥18 years) diagnosed with RRMS who were treated with one of the investigational DMDs (TFN, DMF, FTY, or CLAD). In the case of patients who were prescribed more than one DMD during the period of study, only the oral drug with the longest duration of use was selected for analysis.

The minimum required sample size of 341 patients was determined using the most recent epidemiological data on multiple sclerosis in Lithuania [14] and Cochran’s formula (z = 1.96, for a confidence level of 95% (*p* = 0.05)).

**Figure 1:**
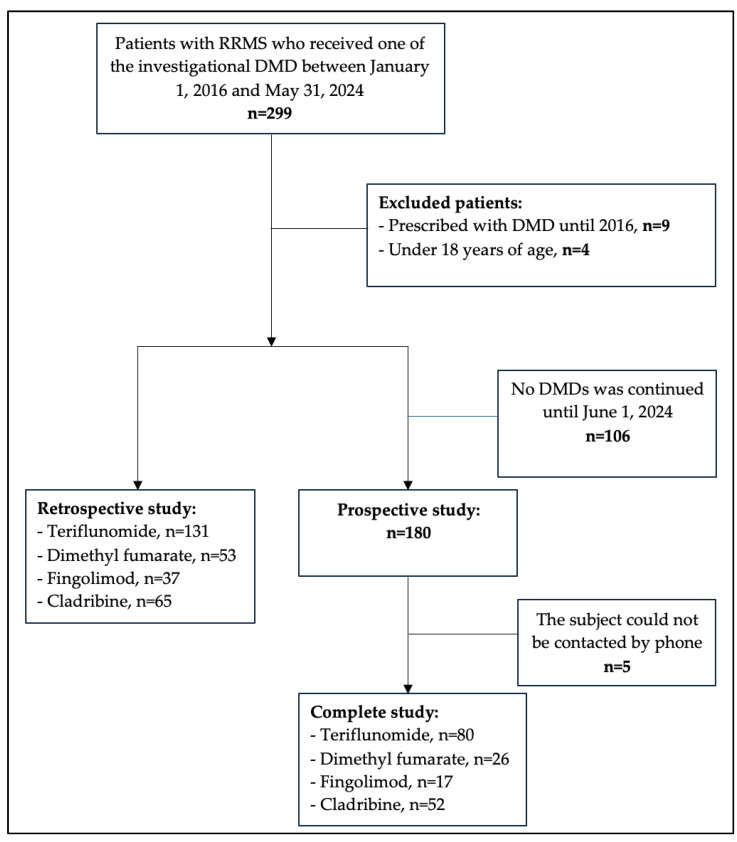
Flowchart of individuals with multiple sclerosis included in the study. RRMS—relapsing–remitting multiple sclerosis; DMD—disease-modifying drug.

The medical records of 299 patients with RRMS registered in the Outpatient Unit of the Department of Nervous System Diseases until 31 May 2024 were reviewed in the retrospective study. Further analysis excluded 9 patients who were prescribed DMDs before 2016 and 4 patients who were prescribed treatment before the age of 18 years, thus reducing the sample size to 286 subjects. The prospective study involved a total of 180 patients who received and continued treatment with at least one DMD between 1 June 2024 and 30 November 2024, with the consent of the subjects. Out of the total number of patients enrolled in the study, 5 could not be reached by phone using the contact numbers provided; therefore, the final number of subjects in the prospective study was 175 (Figure 1).

### 2.3. Data Analysis

IBM SPSS Statistics © version 29.0.0.0.0 and Microsoft © Excel 2023 version 16.89.1 were used for data analysis. The frequencies of the qualitative (categorical) variables were expressed as absolute values (n) and percentages (%). The differences in frequencies between the groups were verified using the Chi^2^ criterion with a z-value (considered significant when z < −1.96 or z > 1.96).

The threshold for statistical significance (α) was set at 5% or lower (*p* < 0.05). The homogeneity of quantitative variables such as mean age and mean duration of illness in years was tested with ANOVA statistical test with a *post hoc* Tukey test.

Since the baseline characteristics such as age at onset and duration of disease were statistically significantly different between the groups, the ANCOVA statistical test was used to test the hypothesis of equality of the means of the quantitative variables to adjust for age and duration of disease and to nullify the difference in baseline characteristics. A more conservative Bonferroni’s *post hoc* test was chosen as a paired test.

## 3. Results

### 3.1. Demographic and Clinical Characteristics of Patients

The mean age at the onset of RRMS varied from 28.62 years (SD 8.27) in the CLAD group to 37.65 years (SD 10.77) in the TFN group. The mean age at disease onset in the TFN group was significantly higher (37.65 years) than the mean age in the other groups (*p* < 0.001). The proportion of women was higher than the proportion of men in all treatment groups. No significant difference was observed between the gender frequency distributions in the population. The disease duration in years was significantly lower (8.05 years) in the CLAD group compared with all other groups of patients (*p* < 0.001). The mean pre-treatment EDSS was significantly higher in the FTY group (3.4 points) as compared with the DMF group (2.2 points) (*p* = 0.003) (Table 1).

### 3.2. Last Prescription of Disease-Modifying Drug Prior to the Investigational Oral Drug

Interferon beta-1a was the most frequently prescribed last DMD in all groups (with 39 patients (29.8%) in the TFN group, 26 (49.1%) in the DMF group, 10 (27.0%) in the FTY group, and 16 (24.6%) in the CLAD group). The second most frequently prescribed prior drug in the TFN, DMF, and FTY groups was Glatiramer acetate (24 (18.3%), 12 (22.6%), and 8 (21.6%), respectively). In the CLAD group, the second most frequently prescribed prior drug was TFN 12 (18.5%). No prior DMD therapy was administered to 81 patients (28.3%), and the DMF group accounted for the lowest percentage (9.4%) (Table 2).

### 3.3. The Effect of Disease-Modifying Therapies on Disease Progression

The increase in EDSS over time was observed in all drug groups over the entire study period. A significant difference in the mean EDSS was found between the four drug groups over all periods (*p* < 0.05). The mean pre-treatment EDSS was significantly higher in the FTY group (3.4 points) than in the DMF group (2.2 points) (*p* = 0.003). After 1 year, the mean EDSS was significantly higher in the TFN (2.7 points) and FTY (3.4 points) groups compared with the DMF group (2.2 points) (*p* = 0.002). After 2 years, the mean values of the TFN (2.8 points) and FTY groups (3.5 points) were higher than those of the DMF group (2.1 points) (*p* = 0.005). After 3 years, the EDSS was significantly higher in patients treated with FTY (3.7 points) and CLAD (3 points) than in patients treated with DMF (2.2 points) (*p* = 0.001), and after 4 years, the EDSS was significantly higher in patients treated with TFN (3.0 points), FTY (3.8 points), and CLAD (3.0 points) mean scores were significantly higher than those of the DMF group (2.4 points) (*p* < 0.001). The EDSS deterioration was lowest in the DMF group (mean difference: +0.2 points) compared with the other three DMDs (mean difference: +0.4 points) (Table 3).

No significant difference in the mean EDSS was observed between the first- and second-line drug groups in the first year prior to DMD (*p* = 0.070), but it was slightly higher in the second-line drug group (FTY and CLAD). In the first year following the prescribed treatment, the mean EDSS score was higher for patients receiving second-line drugs (*p* = 0.124). In the second, third, and fourth years, a significant difference was observed between the first- and second-line drugs: higher mean EDSS scores were observed in patients receiving FTY and CLAD (*p* = 0.024, *p* = 0.015, and *p* = 0.002, respectively, in the second, third, and fourth years) (Table 4).

Regarding the annual exacerbation rate, the largest difference in exacerbation reduction between the first year prior to treatment and after 4 years per patient annually was observed in the CLAD group (absolute difference −2.21), with a slightly smaller difference in the FTY group (−1.75) and in the TFN group (−1.42). An increase in the annual exacerbation rate was observed in the DMF group (Table 5).

Comparing the absolute reduction in exacerbation rate between the first-line and second-line drugs, the absolute reduction in exacerbation rate was twice as high (−2.00) in the second-line group (Table 6).

The analysis of the effects of DMDs on the development of new lesions in brain MRI revealed no significant difference between the groups in the first and second years. Looking at the dynamics of the development of new lesions, a decrease was observed in the FTY (36.4% in the first year and 15.8% in the second year) and CLAD (44.6% in the first year and 20.8% in the second year) groups, while a moderate increase was observed in the TFN group (25.0% in the first year and 26.4% in the second year) and in the DMF group (32.4% in the first year and 38.7% in the second year) (Table 7).

No significant difference was observed between the contrast-enhancing lesions in the four groups. A decrease in contrast-enhancing lesions was observed in FTY (from 9.1% in year 1 to 5% in year 2) and CLAD (from 16.1% in year 1 to 4.3% in year 2) groups (Table 8).

No significant difference was observed between first- and second-line drugs when comparing the development of new lesions on brain MRI. In the first year of DMD therapy, more new lesions were observed in the second-line group than in the first-line group (42.3% and 27.5%, respectively). In the second year, more new lesions were observed among first-line drug patients (Table 9).

In the first year of DMD therapy, more contrast-enhancing lesions were observed in the second-line group than in the first-line group (8% and 3.9%, respectively). In the second year, more new lesions were observed within the first-line group (Table 10).

### 3.4. Assessment of Adherence to DMD Therapy

No doses of the drug were missed significantly more often in patients receiving TFN (68.8%; z = 3.4) and less often in patients receiving DMF (30.8%; z = −3.1) between 1 June 2024 and 30 November 2024 (6-month period). At least one dose was missed significantly more often in patients receiving DMF (69.2%; z = −3.1) and less often in patients receiving TFN (31.3%; z = −3.4). The distribution among FTY patients was almost even, with 47.1% who did not miss a dose (z = −1.0) and 52.9% who missed at least one dose (z = 1.0), but this distribution was not significant. Patients taking CLAD completed the entire prescribed course of treatment (100%) (Table 11).

Forgetfulness was one of the most frequent reasons reported by patients for non-adherence to drug therapy in the DMF group (26.4%) and slightly less in the TFN (14.5%) and FTY (13.5%) groups. Also, individual cases were associated with infection, fever, gastritis, tick-borne encephalitis vaccine, and diarrhea. Allergic reactions were reported only in the DMF group (Table 12).

### 3.5. Effects of DMDs on Absolute Lymphocyte Counts

**Figure 2:**
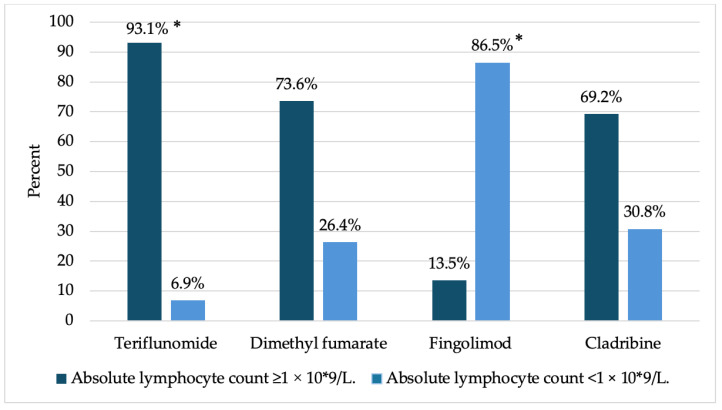
Percentage of patients who developed lymphopenia. * *p* < 0.001.

Lymphopenia was statistically significantly less frequent among TFN patients (93.1%; z = 6.8) and statistically significantly more frequent among FTY patients (86.5%; z = 8.9) (*p* < 0.001). No significant difference was observed between DMF and CLAD, with a higher proportion of patients who did not develop lymphopenia (73.6% and 69.20%, respectively) (Figure 2).

### 3.6. Side Effects Caused by DMD Therapy

The most common side effect reported was lymphopenia caused by DMD (23.77%). The highest proportion of lymphopenia was observed in the FTY group (76.19%). The second most frequent side effects among all patients were diarrhea, skin flushing and itching, and liver enzyme elevations (2.09%, 1.74%, and 1.39%, respectively) in those who received TFN and DMF (Table 13).

## 4. Discussion

A retrospective and prospective study, the first study in Lithuania to evaluate the efficacy, safety, and adherence to oral drug therapy in patients with RRMS, was conducted. Comparison of the demographic characteristics of four investigational DMDs revealed that the duration of MS in years in the CLAD group was significantly the lowest (mean 8.5 years), while the age at onset was statistically significantly the lowest in the FTY and CLAD groups (27.24 and 28.62 years, respectively). At 3 and 4 years after the start of treatment with the investigational DMD, the EDSS was statistically significantly higher in the CLAD, FTY, and TFN group than in the DMF group, and at 2, 3, and 4 years, the EDSS was significantly higher in the second-line groups (FTY and CLAD); the absolute difference in the ARR at 4 years was the highest in CLAD group compared with the other four drugs and the second-line group. CLAD was the second most frequently prescribed first-line DMD (27.7%) in percentage among the groups. In Lithuania, the treatment of multiple sclerosis with CLAD was introduced and added to the list of reimbursable medicines in 2019. Therefore, in the case of a severe form of the disease, predicting a severe course of the disease, it is introduced as a primary drug; thus, the EDSS of the analyzed patient group is high, the age when the drug was prescribed is young, and the duration of the disease is shorter. A study by Spelman et al. comparing the efficacy of CLAD with TFN, DMF, and FTY demonstrated a statically significant lower ARR among patients who received CLAD with the highest EDSS score at the start of the therapy [15]. A retrospective study by Broenlee et al. that compared the efficacy of high-potency (CLAD and FTY) oral drugs over a 9-month period with a full 12-week treatment baseline period showed that CLAD had a higher efficacy rate compared with FTY in terms of ARR [16].

An increase in the ARR was observed in the group of patients taking DMF, while the change in EDSS in this group was significantly the lowest over the four years. This may have been caused by the fact that the change in EDSS was only assessed every single year. The relapses in the DMF group were successfully controlled with high-dose intravenous methylprednisolone therapy; therefore, although the EDSS score was higher during the relapse, in most cases the EDSS score returned to the baseline value after the therapy. Considering prior treatment received, it accounted for the smallest proportion (9.4%) in the group of patients who received DMD before the investigational oral drug, and therefore, the drug was the least frequent choice as the first treatment of MS among the four patient groups. The age at onset of MS was significantly higher in the first-line TFN group (37.65 years), representing the highest proportion of patients who had not received any other DMD therapy, and the absolute difference in ARR was greater in the TFN group compared with the DMF group. The fact that the median age at the onset of multiple sclerosis in the TFN group was the highest among the four drugs may have been associated with the drug being prescribed as a first-line treatment at an older age because of better tolerability and lower risk of infection. The study by Buron et al. evaluating the efficacy of TFN compared with DMF showed a lower ARR in DMF patients, while a meta-analysis by Prosperini et al. also observed a lower ARR in those receiving DMF [17,18]. The Muller study comparing the changes in EDSS between the two drugs revealed a longer time to worsening of EDSS in patients receiving DMF, while no significant difference was found in the study by Laplaud et al. [19,20]. Meanwhile, in our study, the increase in the ARR (i.e., the number of exacerbations increasing over the 4-year dynamics) in the DMF group, with the lowest change in EDSS, indicated that the disease progressed slowly during the treatment period, with milder exacerbations and good control. Calculations showed that EDSS gradually increased over the entire study period, despite the use of disease-modifying therapy, which could be related to progression independent of relapse activity, as observed in the article by Fred D. Lublin et al. [21].

A decrease in the number of new lesions was observed in all groups on T2-weighted brain MRI scans, except for a slight increase in the TFN group, but no significant difference was found. A study by Kalincik et al. revealed that the number of new lesions on T2-weighted scans was lower in FTY group compared with TFN and DMF [22]. A study by Laplaud et al. found no significant difference in the number of new lesions between DMF and TFN groups [20]. A study by Broenlee et al., comparing the changes in MRI contrast-enhancing lesions between CLAD and FTY groups, revealed no significant differences (8% and 7.4%, respectively) [16]. Other studies observed reductions in contrast-enhancing lesions in T1 MRI scans between 85 and 92% over 2 years with CLAD and FTY [23,24,25].

Some of the conducted studies show that improper adherence to the drug therapy has negative effects on the clinical and economic course of the MS patients, including higher numbers of disease exacerbations, faster disease progression, more frequent hospitalizations, and greater financial burden for the health system [26]. In a retrospective analysis of data from the French National Health Service by Vermersch et al., including outpatients and hospitalized patients with RRMS, first-line DMD drugs (TFN or DMF) had better adherence compared with injectable drugs (interferon or Glatiramer acetate) [8]. Other studies assessed adherence to the drug use depending on the dosing regime and revealed that better adherence was associated with DMDs administered once daily (TFN and FTY) than twice daily (DMF) [27]. Our study also revealed that patients receiving DMF had missed at least one dose significantly more frequently and less frequently than patients receiving TFN. Patients taking CLAD completed the entire prescribed course of treatment. Forgetfulness was one of the most frequent reasons reported by patients for non-adherence to drug therapy, while other individual cases included infection, fever, or gastritis. Forgetfulness and side effects of the drug were reported in other clinical trials as well [28].

Lymphopenia was significantly less frequent in patients taking TFN (6.9%) and significantly more frequent in patients taking FTY (86.5%). In the CLAD and DMF groups, lymphopenia was reported in 30.8% and 26.4% of cases, respectively. Our study also revealed that one of the most common side effects of the drug used across all drug groups was lymphopenia (23.77%). By acting through S1P1 receptors, FTY inhibited the exit and entry of lymphocytes from the lymph nodes into the peripheral bloodstream and the entry of abnormal lymphocytes into the central nervous system, resulting in a marked decrease in their number [29]. In other studies, the most significant decrease in the lymphocyte count, up to 70–80% after the start of treatment, was observed in FTY patients, while a decrease in lymphocyte count of up to 25% was observed in CLAD patients within the first two months of treatment and up to 30% in DMF patients within the first year of treatment [11]. Other side effects caused using TFN and DMF identified in our study were associated with in the gastrointestinal system (diarrhea and liver enzyme elevations). Several studies also showed that side effects like diarrhea, nausea, and liver enzyme elevations may be experienced with the use of TFN, and digestive system disorders, lymphopenia, and redness of the skin may be experienced with the use of DMF [30,31].

One of the shortcomings of the study is that the minimum sample size was not met. Some patients were excluded from the study due to the defined analysis period. A significant number of data were not collected due to the lack of the data in the medical documentation (follow-up brain MRI scans of some patients were performed different times) and the absence of the necessary data whether the drug was previously discontinued or replaced with another without reaching the time reference points defined in the study over the treatment period in the defined reference periods for the analyzed indicators (ARR, EDSS, and MRI).

However, most of the results obtained during the study of the real-life clinical practice were consistent with those reported in previous studies comparing the data of patients taking TFN, DMF, FTY, and CLAD. The efficacy, safety, and adherence of DMDs were evaluated in a routine care setting. Second-line drugs were considered for administration in cases of severe course of disease or when the performed tests suggested a severe course of disease. Potentially inconvenient administration of DMF (used twice daily) may lead to skipping of doses, which could make it more difficult to control the course of the disease. FTY-induced lymphopenia could weaken the immune system and result in increased exposure to infectious diseases, which would worsen tolerability of the drug and exacerbate the symptoms of the underlying disease. Notably, the study enabled the creation of a real-world database that can be expanded and used to support future research.

## 5. Conclusions

Over four years, second-line patients had greater ARR reduction, fewer MRI lesions, and higher EDSS from year two. DMF showed the lowest adherence, mainly due to patient forgetfulness, while lymphopenia occurred most frequently with FTY.

## Figures and Tables

**Table 1:** Demographic and clinical characteristics of patients.

Demographic and Clinical Characteristics	Teriflunomide (n = 131)	Dimethyl Fumarate (n = 53)	Fingolimod (n = 37)	Cladribine (n = 65)
Age at disease onset (years) (SD)	37.65 (10.77) *	30.89 (9.57)	27.24 (9.22)	28.62 (8.27)
Gender, n (%)				
Men	48.0 (36.6)	25.0 (47.2)	16.0 (43.2)	20.0 (30.8)
Women	83.0 (63.4)	28.0 (52.8)	21.0 (56.8)	45.0 (69.2)
Disease duration (years) (SD)	12.69 (7.23)	11.60 (5.90)	13.16 (6.26)	8.5 (5.62) *
Mean EDSS (SD) ***	2.6 (1.1)	2.2 (0.9) **	3.4 (1.3) **	2.6 (0.6)
ARR ****	1.477	1.566	1.857	2.213

SD—standard deviation; EDSS—expanded disability status scale; ARR—annualized relapse rate. * *p* < 0.001. ** *p* = 0.003. *** EDSS 1 year before administration of the disease-modifying drug. **** ARR 1 year before administration of the disease-modifying drug.

**Table 2:** Last disease-modifying therapy prior to the investigational oral drug.

Last DMD Used	Teriflunomide (n = 131)	Dimethyl Fumarate (n = 53)	Fingolimod (n = 37)	Cladribine (n = 65)	Total
Dimethyl fumarate, n (%)	0 (0.0)	0 (0.0)	5 (13.5)	4 (6.2)	9 (3.1)
Interferon beta-1a, n (%)	13 (9.9)	6 (11.3)	1 (2.7)	0 (0.0)	20 (7.0)
Fingolimod, n (%)	0 (0.0)	0 (0.0)	0 (0.0)	9 (13.8)	9 (3.1)
Glatiramer acetate, n (%)	24 (18.3)	12 (22.6)	8 (21.6)	3 (4.6)	47 (16.4)
Interferon beta-1a, n (%)	39 (29.8)	26 (49.1)	10 (27.0)	16 (24.6)	91 (31.8)
Laquinimod, n (%)	3 (2.3)	0 (0.0)	0 (0.0)	0 (0.0)	3 (1.0)
Natalizumab, n (%)	0 (0.0)	0 (0.0)	2 (5.4)	0 (0.0)	2 (0.7)
Ocrelizumab, n (%)	0 (0.0)	0 (0.0)	0 (0.0)	1 (1.5)	1 (0.3)
Ponesimod, n (%)	0 (0.0)	0 (0.0)	0 (0.0)	2 (3.1)	2 (0.7)
Teriflunomide, n (%)	0 (0.0)	4 (7.5)	5 (13.6)	12 (18.5)	21 (7.3)
No prior disease-modifying therapy was prescribed, n (%)	52 (39.7)	5 (9.4)	6 (16.2)	18 (27.7)	81 (28.3)

DMD—disease-modifying drug.

**Table 3:** Change in EDSS before and after DMD therapy in different groups.

Change in EDSS	Teriflunomide	Dimethyl Fumarate	Fingolimod	Cladribine	Significance
1 year before treatment					
N	131	53	37	65	*p* = 0.003
Mean score (SD)	2.6 (1.1)	2.2 (0.9) *	3.4 (1.3) *	2.6 (0.6)	
Year 1 of treatment					
N	120	52	36	56	*p* = 0.002
Mean score (SD)	2.7 (1.1) *	2.2 (1.0) *	3.4 (1.1) *	3.1 (1.5)	
Year 2 of treatment					
N	106	44	34	32	
Mean score (SD)	2.8 (1.1) *	2.1 (0.9) *	3.5 (1.1) *	3.1 (1.4)	*p* = 0.005
Year 3 of treatment					
N	81	35	28	32	*p* = 0.001
Mean score (SD)	2.9 (1.1)	2.2 (1.1) *	3.7 (1.2) *	3.0 (1.3) *	
Year 4 of treatment					
N	67	24	27	4	
Mean score (SD)	3.0 (1.2) *	2.4 (1.2) *	3.8 (1.1) *	3.0 (1.3) *	*p* < 0.001
Mean difference between the first year before and after 4 years of DMD	+ 0.4	+ 0.2	+ 0.4	+ 0.4	-

EDSS—expanded disability status scale; DMD—disease-modifying drug; SD—standard deviation. * *p* < 0.05, Bonferroni’s paired test.

**Table 4:** Comparison of the change in EDSS between first- and second-line DMDs at different time periods.

Change in EDSS	First Line	Second Line	Significance
Year 1 before treatment—mean score (SD)	2.55 (1.1)	2.75 (1.3)	*p* = 0.07
Year 1 of treatment—mean score (SD)	2.64 (1.1)	2.81 (1.2)	*p* = 0.124
Year 2 of treatment—mean score (SD)	2.73 (1.1)	3.09 (1.2)	*p* = 0.024
Year 3 of treatment—mean score (SD)	2.79 (1.2)	3.94 (1.2)	*p* = 0.015
Year 4 of treatment—mean score (SD)	2.81(1.2)	3.6 (1.2)	*p* = 0.002

EDSS—expanded disability status scale; DMD—disease-modifying drug; SD—standard deviation.

**Table 5:** A breakdown of the annual exacerbation rates at different time periods in the four drug groups.

Annualized Relapse Rate	Teriflunomide (n = 131)	Dimethyl Fumarate (n = 53)	Fingolimod (n = 37)	Cladribine (n = 65)
Year 1 before treatment	1.477	1.566	1.857	2.213
Year 1 of treatment	0.150	1.094	1.086	1.389
Year 2 of treatment	0.115	1.239	1.206	0.135
Year 3 of treatment	0.158	1.487	0.138	0.182
Year 4 of treatment	0.058	1.697	0.103	0.000
Absolute difference	−1.42	0.13	−1.75	−2.21

**Table 6:** A breakdown of the annual exacerbation rate at different time periods between the first-line and second-line drugs.

Annualized Relapse Rate	First-Line	Second-Line
Year 1 before treatment	1.50	2.08
Year 1 of treatment	0.44	1.27
Year 2 of treatment	0.44	0.65
Year 3 of treatment	0.54	0.79
Year 4 of treatment	0.51	0.08
Absolute reduction in exacerbation rate	−0.99	−2.00

**Table 7:** Changes in brain MRI T2 during DMD therapy by year between the four groups.

MRI T2 Changes	Teriflunomide	Dimethyl Fumarate	Fingolimod	Cladribine	Significance
Year 1, n (%)					
No changes	51 (75.0)	23 (67.6)	14 (63.6)	31 (55.4)	*p* = 0.146
≥1 new lesion	17 (25)	11 (32.4)	8 (36.4)	25 (44.6)	
Year 2, n (%)					
No changes	53 (73.6)	19 (61.3)	16 (84.2)	19 (79.2)	*p* = 0.276
≥1 new lesion	19 (26.4)	12 (38.7)	3 (15.8)	5 (20.8)	

MRI—magnetic resonance imaging; DMD—disease-modifying drug.

**Table 8:** Changes in brain MRI T1 during DMD therapy by year between the four groups.

New Contrast-Enhancing Lesions in T1 MRI	Teriflunomide	Dimethyl Fumarate	Fingolimod	Cladribine	Significance
Year 1, n (%)					
Non-enhancing lesions	65 (95.6)	34 (97.1)	20 (90.9)	47 (83.9)	*p* = 0.066
Enhancing lesions	3 (4.4)	1 (2.9)	2 (9.1)	4 (16.1)	
Year 2, n (%)					
Non-enhancing lesions	65 (90.3)	30 (96.8)	19 (95)	22 (95.7)	*p* = 0.593
Enhancing lesions	7 (9.7)	1 (3.2)	1 (5)	1 (4.3)	

MRI—magnetic resonance imaging; DMD—disease-modifying drug.

**Table 9:** Changes in brain MRI T2 while receiving DMD therapy by year between the first-line and second-line groups.

MRI Changes in T2 Mode	First Line	Second Line	Significance
Year 1, n (%)			
No changes	74 (72.5)	45 (57.7)	*p* = 0.501
≥1 new lesion	28 (27.5)	33 (42.3)	
Year 2, n (%)			
No changes	72 (69.9)	35 (81.4)	*p* = 0.153
≥1 new lesion	31 (30.1)	8 (18.6)	

MRI—magnetic resonance imaging; DMD—disease-modifying drug.

**Table 10:** Changes in brain MRI T1 while receiving DMD therapy by year between the first-line and second-line groups.

New Contrast-Enhancing Lesions in T1 MRI	First Line	Second Line	Significance
Year 1, n (%)			
Non-enhancing lesions	99 (96.1)	69 (92)	*p* = 0.235
Enhancing lesions	4 (3.9)	6 (8)	
Year 2, n (%)			
Non-enhancing lesions	95 (92.2)	41 (95.3)	*p* = 0.497
Enhancing lesions	8 (7.8)	2 (4.7)	

MRI—magnetic resonance imaging; DMD—disease-modifying drug.

**Table 11:** Administration of the entire course of DMD therapy over a 6-month period per drug.

Missed Doses Within 6 Months	Teriflunomide (n = 80)	Dimethyl Fumarate (n = 26)	Fingolimod (n = 17)	Cladribine (n = 52)	Significance
No, n (%)	55 (68.8) (z = 3.4)	8 (30.8) (z = −3.1)	8 (47.1) (z = −1.0)	52 (100)	*p* = 0.005
Yes, n (%)	25 (31.3) (z = −3.4)	18 (69.2) (z = 3.1)	9 (52.9) (z = 1.0)	0 (0)	

DMD—disease-modifying drug.

**Table 12:** A breakdown of reasons for non-adherence by drug.

Reasons for Non-Adherence	Teriflunomide (n = 80)	Dimethyl Fumarate (n = 26)	Fingolimod (n = 17)	Cladribine (n = 52)	Total (n = 175)
Not included in the analysis, N (%)	106 (80.9)	34 (64.2)	28 (75.7)	65 (100)	233
Allergic reaction, N (%)	0 (0.0)	2 (3.8)	0 (0.0)	0 (0.0)	2 (0.7)
Alcohol consumption, N (%)	1 (0.8)	0 (0.0)	0 (0.0)	0 (0.0)	1 (0.3)
Vaccination against encephalitis, N (%)	0 (0.0)	0 (0.0)	1 (2.7)	0 (0.0)	1 (0.3)
Infection, N (%)	2 (1.5)	1 (1.9)	2 (5.4)	0 (0.0)	5 (1.7)
Fever, N (%)	1 (0.8)	0 (0.0)	0 (0.0)	0 (0.0)	1 (0.3)
Gastritis, N (%)	0 (0.0)	1 (1.9)	0 (0.0)	0 (0.0)	1 (0.3)
Lymphopenia, N (%)	0 (0.0)	0 (0.0)	1 (2.7)	0 (0.0)	1 (0.3)
Failed to purchase the drug, N (%)	1 (0.8)	1 (1.9)	0 (0.0)	0 (0.0)	2 (0.7)
Forgetfulness, N (%)	19 (14.5)	14 (26.4)	5 (13.5)	0 (0.0)	38 (13.3)
Diarrhea, N (%)	1 (0.8)	0 (0.0)	0 (0.0)	0 (0.0)	1 (0.3)

**Table 13:** Distribution of side effects caused by DMD therapy.

Side Effects	Teriflunomide (n = 131)	Dimethyl Fumarate (n = 53)	Fingolimod (n = 37)	Cladribine (n = 65)	All Four Drugs (n = 286)
No side effects experienced, n (%)	111 (84.73)	33 (62.24)	4 (10.81)	45 (69.23)	193 (67.48)
Lymphopenia, n (%)	8 (6.11)	11 (20.75)	32 (86.48)	17 (26.15)	68 (23.77)
Herpes rash on the lips, n (%)	0 (0.0)	0 (0.0)	0 (0.0)	1 (1.53)	1 (0.34)
Subcutaneous bruises, n (%)	0 (0.0)	0 (0.0)	0 (0.0)	1 (1.53)	1 (0.34)
Liver enzyme elevation, n (%)	3 (2.29)	0 (0.0)	0 (0.0)	1 (1.53)	4 (1.39)
Cardiovascular adverse events (hypertension and tachycardia), n (%)	0 (0.0)	0 (0.0)	1 (2.70)	0 (0.0)	1 (0.34)
Redness, itching of the skin following ingestion, n (%)	0 (0.0)	5 (9.43)	0 (0.0)	0 (0.0)	5 (1.74)
Diarrhea, n (%)	5 (3.81)	1 (1.88)	0 (0.0)	0 (0.0)	6 (2.09)
Abdominal pain, n (%)	0 (0.0)	3 (5.66)	0 (0.0)	0 (0.0)	3 (1.04)
Suspected rectal cancer, n (%)	1 (0.76)	0 (0.0)	0 (0.0)	0 (0.0)	1 (0.34)
Hair fall, n (%)	2 (1.52)	0 (0.0)	0 (0.0)	0 (0.0)	2 (0.69)
Purulent conjunctivitis, n (%)	1 (0.76)	0 (0.0)	0 (0.0)	0 (0.0)	1 (0.34)

## Data Availability

Data are contained within the article.

## Abbreviations

MS	Multiple sclerosis
RRMS	Relapsing–remitting multiple sclerosis
RR	Relapsing–remitting
TFN	Teriflunomide
DMF	Dimethyl fumarate
FTY	Fingolimod
CLAD	Cladribine
DMDs	Disease-modifying drugs
DMD	Disease-modifying drug
MRI	Magnetic resonance imaging
EDSS	Expanded disability status scale
ARR	Annualized relapse rate
NF-E2	Nuclear Factor-Erythoid 2
